# Supplementary material for: Nutritional status, environmental enteric dysfunction, and prevalence of rotavirus diarrhoea among children in Zambia
Source: PLoS One. 2020 Oct 2;15(10):e0240258. doi: 10.1371/journal.pone.0240258 (PMC7531814; doi:10.1371/journal.pone.0240258)
Supplement: Attachment — Submitted filename: Responses to Reviewer Comments.docx [file pone.0240258.s001.docx]

**Title:** Nutritional Status, Environmental Enteric Dysfunction, and Prevalence of Rotavirus Diarrhoea Among Children in Zambia

**Comments to the Author**

Please use the space provided to explain your answers to the questions above. You may also include additional comments for the author, including concerns about dual publication, research ethics, or publication ethics. (Please upload your review as an attachment if it exceeds 20,000 characters)

Reviewer #1: Koyuncu et al. expand on a study performed from 2012-2013 in children in Zambia to determine whether environmental enteric dysfunction impacts rotavirus infection. The authors report that morphological changes that likely result from environmental enteric dysfunction may paradoxically provide protection against rotavirus infection in children. The manuscript is thought provoking, well written and provides novel findings. The manuscript could benefit from more discussion on potential scientific mechanisms that may provide the protection, I feel that this would strengthen the manuscripts findings. Overall, the authors’ background, methods, findings and conlcusions are logical, well laid out and compelling.

Minor Comments:
1. Please define LMIC (line 74)

Thank you for noting this, the correction has been made.

2. I think table 1 would be easier to read if the characteristics were bolded or underlined to separate out the characteristics ie: bold or underline sex and then male female would be easier to identify as separate from the next characteristic Child age in years. A structure similar to table 2 with characteristics separated with lines would also work well.

Thank you for this suggestion, we have updated table 1 accordingly.

3. Line 202 different should read differ.

Thank you, the correction has been made.

4. Please elaborate in the discussion on why morphological changes in the gut and inflammation may impact the capability of rotavirus to invade the gastrointestinal tract. Are there any scientific papers that suggest that villous blunting diminishes rotavirus infection or whether increased cell shedding or altered microbiota in malnourished children contributes to decreased rotavirus impact? If so, these studies would be helpful to discuss and reference and would provide additional support for the authors’ conclusions.

Thank you for the opportunity to elaborate. The discussion section has been amended to include the following:

“The morphological changes associated with EED develop in response to chronic episodes of enteric infection in settings with inadequate WASH. In settings with persistent exposure to faecal pathogens, it is plausible that the pro-inflammatory and hyperactivated immune states associated with EED may develop as the body’s defense to future pathogenic infiltration [11,22]. Malnutrition and constant exposure to fecal contamination is also hypothesized to influence the composition of gut-microbiota, which may influence susceptibility to rotavirus [8,11]. For example, in a study conducted by Kuss et al antibiotic depletion of gut microbiota in mice was associated with a reduced ability of polio viruses to infect host cells in the small intestine [23]. Although the effects of EED on microbial ecology are poorly understood, alterations in the composition of commensal bacteria may mediate the association between malnutrition and susceptibility to rotavirus infection.”
